# Pediatric Epidemic of *Salmonella enterica* Serovar Typhimurium in the Area of L’Aquila, Italy, Four Years after a Catastrophic Earthquake

**DOI:** 10.3390/ijerph13050475

**Published:** 2016-05-06

**Authors:** Giovanni Nigro, Gabriella Bottone, Daniela Maiorani, Fabiana Trombatore, Silvana Falasca, Gianfranco Bruno

**Affiliations:** 1 Pediatric Unit, University of L’Aquila, San Salvatore Hospital, L’Aquila 67100, Italy; gabriellabottone@gmail.com (G.B.); daniela.maiorani82@gmail.com (D.M.); fabiana_t@alice.it (F.T.); 2 Department of Clinical Pathology, San Salvatore Hospital, L’Aquila 67100, Italy; silvana.falasca@alice.it (S.F.); supergiaf@gmail.com (G.B.)

**Keywords:** *Salmonella enterica* serovar typhimurium, *Salmonella* epidemic, *Salmonella* reinfection, water survey stations

## Abstract

*Background*: A *Salmonella enterica* epidemic occurred in children of the area of L’Aquila (Central Italy, Abruzzo region) between June 2013 and October 2014, four years after the catastrophic earthquake of 6 April 2009. *Methods*: Clinical and laboratory data were collected from hospitalized and ambulatory children. Routine investigations for Salmonella infection were carried out on numerous alimentary matrices of animal origin and sampling sources for drinking water of the L’Aquila district, including pickup points of the two main aqueducts. *Results*: Salmonella infection occurred in 155 children (83 females: 53%), aged 1 to 15 years (mean 2.10). Of these, 44 children (28.4%) were hospitalized because of severe dehydration, electrolyte abnormalities, and fever resistant to oral antipyretic and antibiotic drugs. Three children (1.9%) were reinfected within four months after primary infection by the same Salmonella strain. Four children (2.6%), aged one to two years, were coinfected by rotavirus. A seven-year old child had a concomitant right hip joint arthritis. The isolated strains, as confirmed in about the half of cases or probable/possible in the remaining ones, were identified as *S. enterica* serovar Typhimurium [4,5:i:-], monophasic variant. Aterno river, bordering the L’Aquila district, was recognized as the main responsible source for the contamination of local crops and vegetables derived from polluted crops. *Conclusions*: The high rate of hospitalized children underlines the emergence of a highly pathogenic *S. enterica* strain probably subsequent to the contamination of the spring water sources after geological changes occurred during the catastrophic earthquake.

## 1. Introduction

*Salmonella enterica* is one of the most common enteric pathogens of humans and animals, which causes over 90 million cases worldwide each year implying considerable illness and economic burden (in the United States >2 billion USD per year) [1]. *S. enterica* consists of >2500 serovars, of which *S. typhimurium* is the most ubiquitous in zoonotic reservoirs for human infection and the environment [2]. Over the past half century, the epidemiology of *S. typhimurium* has been characterized by successive waves of dominant multidrug-resistant clones [3]. During a 2005–2012 epidemic, comparative whole-genome sequencing and phylogenomic analysis of isolates from the United Kingdom and Italy showed high levels of genotypic variation of monophasic *S. typhimurium* affecting antigens, virulence factors, and resistance loci [4].

The monophasic variant of *S. typhimurium*, *S.* 4,[5],12:i:-, characterized by the antimicrobial resistance to Ampicillin, Streptomycin, Sulphonamide, and Tetracycline (pattern ASSuT) is emerging and extensively circulating in Denmark, Italy, United Kingdom, and Greece [4,5,6]. In Italy, *S. typhimurium* 4,[5],12:i:-, showed a dramatic increase since 2003, both in humans and in animals farmed for food production, particularly pigs and bovines [7]. Most serovars showed a marked seasonality, increasing over the summer months and peaking in August/September, which may be related to the parallel *Salmonella* shedding trend in animal hosts, and/or insufficient refrigeration and mishandling of foods during the warm months [8]. Non-typhoid Salmonella epidemics have never been associated with risk of consuming contaminated water following collapse of sewerage systems due to a devastating earthquake. We report about the *S. typhimurium* epidemic which occurred between June 2013 and October 2014 in children of the area of L’Aquila (Central Italy, Abruzzo region) four years after the catastrophic 6.3 magnitude earthquake of 6 April 2009.

## 2. Material and Methods

### 2.1. Patients

Over a period of three months (October–December 2013) the Epidemiological Unit of the San Salvatore hospital, L’Aquila, was alarmed because of an unusual number of children admitted with acute gastroenteritis or enterocolitis caused by Salmonella infections. To coordinate territorial investigation tasks, and to simplify sharing of the collected data, a multidisciplinary work-group was set up including the National Health Institute (Monitoring Network, EnterNet Italy), the Experimental Zoo-prophylactic Institute (Reference Regional Laboratory for Enterobacterial in, Veterinary Operation Centre for Epidemiology, Programming and Information), the ASL 1 Abruzzo (Department of Hygiene, Epidemiology and Public Health; and Department of Food and Nutrition Security). The following definitions were adopted: (a) Epidemic strain: isolated Salmonella strain; (b) Confirmed case: any subject resident in the area of L’Aquila from which the epidemic strain of Salmonella was isolated during the study period; (c) Suspected case: any subject resident in Abruzzo region and/or any subject who stayed in Abruzzo at least seven days before the onset of gastrointestinal symptoms; (d) Probable case: any subject resident in the area of L’Aquila, who was affected by diarrhea (at least three stools/24 h for three consecutive days) from June 2013 to October 2014.

### 2.2. Survey on Food Products and Environmental Matrices

The analyzed alimentary matrices included fresh sausage withdrawn from different food industries, home-made sausage, smoked and cured sausage withdrawn from one food industry, wild boar salami withdrawn from a food industry, ham, chicken/turkey or pork frankfurter withdrawn from kitchen serving school, chicken withdrawn from kitchens serving schools, liquid pasteurized eggs withdrawn from kitchens serving schoosl, fresh eggs, dry cured smoked ham and cured pork mayonnaise, and tap water from private houses. Numerous sampling sources for drinking water of the L’Aquila area were collected from the following sites: pickup points of the two main aqueducts, schools, main kitchens serving schools, private houses.

### 2.3. Microbiological Investigations

The isolated Salmonella strains were identified biochemically by Vitek 2 system (Biomerieux, Marcy-l’Etoile, France) and serologically by commercially available antisera (Statens Serum Institut, Copenhagen, Denmark) following the Kauffmann-White method. The strains were identified by multiplex polymerase chain reaction and typed by phage typing, pulsed-field gel electrophoresis (PFGE), and multiple-locus variable analysis (MLVA) following ECDC laboratory standard operating procedures [9,10]. Genomic DNA was extracted by Qiagen Easy Prep (Foster City, CA, USA), libraries were prepared using the Hi-Q sequencing kit, and sequencing was performed on a PGM Ion Torrent platform [6]. Raw reads were submitted to the SRA repository, and the samples were registered under the project ID PRJNA266093. Reads were trimmed and assembled using a *de novo* workflow under the Orione framework plus some specific python scripts [6].

## 3. Results

### 3.1. Clinical Features

**Figure 1:**
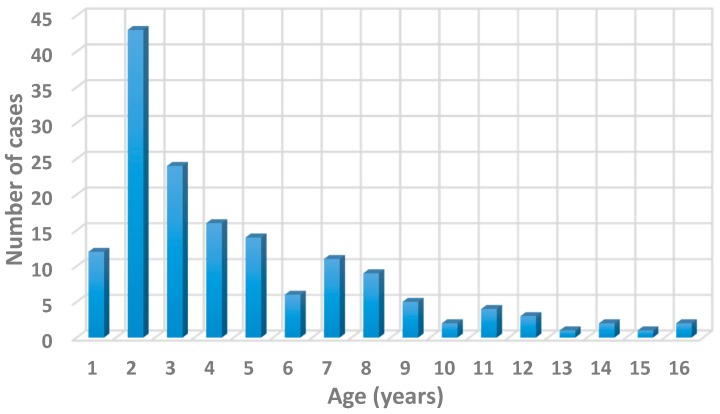
Distribution by age of 155 children with *Salmonella enterica* Typhimurium infection.

The epidemic affected 155 children (83 females: 53%), all but two Italian, aged 1 to 15 years (mean 2.10). The distribution by age showed a prevalence of one to two year-old patients (Figure 1).

**Figure 2:**
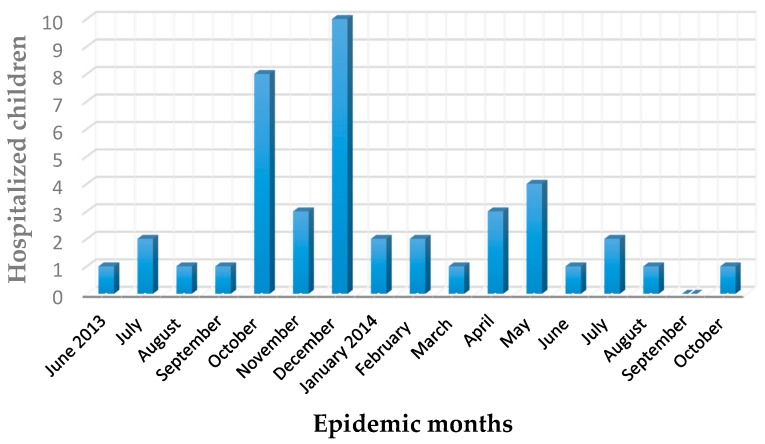
Distribution by months of 44 children with *Salmonella enterica* Typhimurium infection.

Forty-four of the infected children (28.4%) were hospitalized because of diarrhea, blood in almost half of them, severe dehydration, electrolyte abnormalities, vomiting, and fever resistant to oral antipyretic and antibiotic drugs. Three children (1.9%) were reinfected within four months after primary infection by the same Salmonella strain. Four children (2.6%), aged one to two years, were coinfected by rotavirus. A seven-year old child had a concomitant right hip joint arthritis, the origin of which remained unknown because the synovial fluid (non-corpuscular by ultrasound) was not taken. Figure 2 shows the distribution by months of the hospitalized children.

All the remaining children were followed up as outpatients since they had short-term diarrhea, which was associated with slight fever or vomiting in a minority of them (Table 1).

Mean laboratory results showed the occurrence of a slight neutrophilic leukocytosis with decreased levels of sodium (Table 2).

During hospital stay, all children were given intravenous rehydration and intravenous antibiotic (amoxicilline-clavulanic acid in 40% and cephalosporins in 60% of cases) therapy. All children were discharged after confirmation of negative stool samples, and were given oral antibiotic therapy, to prevent reappearance of the symptoms and possible epidemic overspread. 

### 3.2. Epidemiological and Bacteriological Investigations

*S. typhymurium* strains were isolated from samples of surface waters and sewage plants, all obtained in the area of L’Aquila. Serotyping of the isolated strains showed that all of them could be identified as *S. enterica* serovar *Typhimurium* [4,5:i:-], monophasic variant [6]. As reported by Orsini *et al.*, whole-genome sequencing was performed on 19 strains, which were chosen among clinical and environmental isolates because of their spatial-temporal distribution and familiar kinship, and three unrelated strains as outgroups. The genomic results, including numbers of coding sequences (CDSs), rRNAs, and tRNAs, *N*50 values and GenBank accession numbers, have been published [6]. On the basis of the maximum-likelihood phylogenetic tree derived from the Single Nucleotide Polymorphism matrix of sequenced strains, a close relationship between the isolates of water sources and isolates from patients was suggested [11].

Results of the survey showed that all the samples of drinking water and of chicken, veal meat, cooked and dry-cured hams, and eggs, which were the most consumed food one week before the clinical onset of infection, were negative. Other expositions to risk factors in the seven days before the onset of clinical symptoms of epidemic cases included school/nursery attendance, animal contact, exposition to symptomatic relatives, attending a banquet, traveling.

Starting from June 2014, the health authorities banned the use of surface waters for crop irrigation in the area of L’Aquila and decontaminating interventions were performed in water cleaning plants.

## 4. Discussion

*S. typhimurium* is the most reported serovar causing non-typhoidal salmonellosis in Italy since 2000, while *S. enteritidis* is still prevailing in other countries [7]. In immunocompetent individuals of high-income countries, infections by *S. typhimurium* mainly cause a self-limiting enterocolitis including vomiting, profuse watery diarrhea, and abdominal pain [12]. Invasive strains of non-typhoidal salmonellae can produce severe bloodstream infections in immunodepressed adults and malnourished children, as recently occurred during the outbreak caused by a new genotype of *S. typhimurium*, ST313, in the sub-Saharan Africa [13]. However, in both industrialized and developing countries, children aged one to six years have a higher risk of infection by Salmonella species [14]. During the epidemic occurred in the area of L’Aquila, the age risk was lowered to children less than two years of age. This elevated risk does not appear to be related to surveillance bias since clinical pathways (medical consultation, hospitalization, diagnostic tools) are the same for the age group of one to six years. Therefore, the higher risk of acquiring the disease may be due to age-related behavioral factors favoring greater susceptibility to the agent and risk of exposure to the vehicle of infection in domestic environment by cross contamination, particularly when the vehicle is a food for infants. 

Contrary to previous *S. typhimurium* epidemic in Italy and other countries, the symptomatology was frequently severe in many children aged less than two years, and required hospitalization for rehydration and intravenous antibiotic therapy [1,7]. A five-year old boy also had a concomitant hip joint arthritis, which can occasionally occur during *S. enterica* infection [15]. 

Microbiological characteristics of the epidemic strain appear to be unusual, and the lack of correspondence with other strains in the database of human surveillance in Italy and in Europe does not allow the hypothesizing of animals as well as food as potential sources of infection. In fact, descriptive epidemiology suggests that the epidemic source may be represented by a locally produced and distributed vehicle. The potential sources fulfilling the epidemic scenario are the following: (a) long shelf life foods contaminated during the primary production process (e.g., food contaminated in the pre-harvest phase, stored and distributed within many months; (b) primarily contaminated short shelf life foods that steadily enter the market; (c) short shelf life foods that can be contaminated in the site of production environment and constantly enter the market of the Abruzzo region (e.g., food from animal origin such as meat, fruit and vegetables, dairy products). Moreover, the wide geographical origin of epidemic cases suggests that the contamination is not to be referred to a single cause but to a combination of different causes, probably of environmental origin. 

Numerous reports underlined the high grade of contamination in the rivers of the Abruzzo region after the earthquake. Quality of the water of the Aterno river, bordering the L’Aquila district, was classified as “poor quality” and the river pollution was recognized as responsible for contamination of local crops irrigated by its water. Furthermore, as reported by Gordon *et al.* in Malawi, the local high rain rate subsequent to climatic changes lead to the same consequences by inducing the overflow of small effluents and subsequently the contamination of bordering crops [16]. Consequently, the source of infection could be related to ingestion of vegetable stocks prepared from manipulation of vegetables derived from polluted crops. In USA, several Salmonella outbreaks have been associated with irrigation waters as potential sources of contaminated food [17,18,19]. The occurrence of clinical cases due to the same Salmonella strain being isolated also in other regions only in patients who had travelled to L’Aquila could confirm the existence of unique environmental features in this area. Therefore, the initial cause of the epidemic spread may have been the geological change due to the catastrophic earthquake that occurred in the area of L’Aquila on 6 April 2009, and the subsequent contamination of the spring water sources following the system failure of the water cleaning plant collecting sewage. In 2007, Fujimoto *et al.* reported about the oil contamination of the Shinano river water after the Niigata Chuetsu earthquake occurred in 23 October 2004 [20]. To our knowledge, ours is the first report about environmental water contamination by bacteriological pathogens following a catastrophic earthquake.

Finally, the emergence of a highly virulent strain of *S. typhimurium* could be related to the arrival to L’Aquila, since October 2011, of thousands of workers and their relatives from East European countries, who presumably hosted Salmonella strains different from those of people of L’Aquila. This observation may be supported by the hospitalization of only two non-Italian children, who were presumably in part immunized. The presence of different *S. typhimurium* strains during the epidemic may be supported by the occurrence of reinfections in three children.

## 5. Conclusions

In conclusion, our report highlights the emergence of a new highly pathogenic Salmonella strain causing a long term epidemic in the area of L’Aquila four years after a catastrophic event. To prevent serious epidemics consequent to geological reassessment and ecological changes, the water survey stations and sewage systems should be carefully monitored, and wider public health issues through structural changes in urban design should be addressed.

## Figures and Tables

**Table 1:** Clinical findings in 155 children with *Salmonella enterica* Typhimurium infection hospitalized or followed up as outpatients.

Clinical Findings	Hospitalized Patients (44)		Outpatients (111)	
	No.	%	No.	%
*Diarrhea*	44	100	111	100
*Bloody diarrhea*	25	57	5	4.5
*Fever*	44	100	22	19.8
*Dehydration*	42	100	0	0
*Electrolyte imbalance*	16	36	0	0
*Vomiting*	28	64	7	6.3
*Hip joint arthritis*	1	2	0	0
*Hospital stay (days)*	4.1		_	

**Table 2:** Laboratory findings in 44 hospitalized children with *Salmonella enterica* Typhimurium infection, on admission.

Laboratory Findings	Range		Mean Values
	Min	Max	
*Red blood cells (x mm^3^)*	3.39	5.62	4.89
*Hemoglobin (g/dL)*	11.1	15.2	13.0
*White blood cells (x mm^3^)*	5.3	22.3	10.1
*Neutrophils (%)*	24.7	90.4	64.8
*Lymphocytes (%)*	7.8	58.4	23.6
*Unclassified lymphocytes (%)*	0.6	9.9	3.7
*Platelets (x mm^3^)*	213	836	330
*Sodium (mEq/L)*	126	142	133
*Potassium (mEq/L)*	3.4	5.1	4.0
